# The C-Reactive Protein to Albumin Ratio as a Predictor of Severe Side Effects of Adjuvant Chemotherapy in Stage III Colorectal Cancer Patients

**DOI:** 10.1371/journal.pone.0167967

**Published:** 2016-12-08

**Authors:** Tetsuro Tominaga, Takashi Nonaka, Yorihisa Sumida, Shigekazu Hidaka, Terumitsu Sawai, Takeshi Nagayasu

**Affiliations:** 1 Department of Surgical Oncology, Nagasaki University Graduate School of Biomedical Science, Sakamoto, Nagasaki, Japan; 2 Department of cardiopulmonary Rehabilitation Science, Nagasaki University Graduate School of Biomedical Science, Sakamoto, Nagasaki, Japan; Baylor University Medical Center, UNITED STATES

## Abstract

**Background/Aims:**

Adjuvant chemotherapy (AC) has been reported to improve the prognosis for patients with Stage III colorectal cancer (CRC). However, some patients experience severe side effects and must stop AC. The C-reactive protein (CRP) to albumin ratio (CAR) is a novel inflammation-based score that could reflect the patient’s general condition. The aim of this study was to evaluate the predictive value of the CAR for side effects of AC in CRC.

**Methods:**

A total of 136 CRC patients who received AC were retrospectively analyzed. The patients were subdivided into two groups by the CAR level (CAR ≥0.1, n = 30; CD < 0.1, n = 106).

**Results:**

The presence of lymphatic invasion, severe side effects, and discontinuation of AC were associated with high CAR levels (p = 0.02, <0.01, and 0.02; respectively). High levels of the Glasgow Prognostic Score (GPS) and the neutrophil to lymphocyte ratio (NLR) appeared to be associated with the CAR (p = 0.04, p<0.01; respectively). Multivariate analysis identified CAR≥0.1 (HR: 7.06, 95% CI: 2.51–19.88, p<0.01) as a significant determinant of severe side effects of AC. CAR had the highest area under the curve (0.79) among several inflammation-based scores.

**Conclusion:**

The present study showed that the CAR is a novel and promising inflammation-based score for ≥ grade 3 side effects of AC in node-positive CRC.

## Introduction

In the seventh TNM classification, colorectal cancer with lymph node metastasis is defined as Stage III disease [1]. It is reported that about 50% of patients with Stage III cancer have recurrent disease, such as local recurrence and distant metastasis, and a five-year-survival rate of 68–77% [2, 3]. It has been reported that adjuvant chemotherapies (ACs) could result in a 30% decrease of relapse rates compared with surgery alone [4]. Furthermore, several randomized controlled studies have shown that Stage III colon cancer patients have a benefit in terms of both relapse-free survival and overall survival by using combination therapy that includes oxaliplatin [5, 6]. Several studies demonstrated that the recommended standard duration for AC was six months, because it might be a good balance to achieve a good prognosis and minimize the cost [7, 8]. However, there was a constant rate of cases that experienced severe side effects and discontinued AC.

Recently, a number of inflammation-based scores, including the Glasgow Prognostic Score (GPS), the platelet to lymphocyte ratio (PLR), the neutrophil to lymphocyte ratio (NLR), and the Prognostic Nutrition Index (PNI), have been reported to correlate with patient outcome in the field of clinical oncology [9, 10]. A previous study showed that the new inflammation-based score, the CRP/albumin ratio (CAR), was a good predictor of treatment outcomes for patients with hepatocellular carcinoma [11]. In the field of colorectal cancer (CRC), CAR is as useful for predicting the postoperative survival of patients [12]. A high CRP level and a low albumin level could correlate with high inflammation. Chronic and high inflammation means hypercytokinemia that might lead to weight loss and malnutrition. Several studies reported that hypercytokinemia could produce severe side effects during chemotherapy [13–16]. Furthermore, a good nutritional status slows weight loss, stabilizes body composition, and could improve quality of life in patients with advanced CRC, which reduces chemotherapy-associated side effects [17].

The aim of this retrospective study was to evaluate the predictive value of the CAR for the side effects of AC and the prognosis in node-positive CRC patients. Furthermore, the predictive value of the CAR for side effects was compared to those of several inflammation-based scores, such as the GPS, PLR, and NLR.

### Patients

An ethical committee, Nagasaki university hospital approved this retrospective observational study. Written informed consent was obtained from all patients before surgery.

From April 2005 to March 2014, 795 CRC patients underwent colorectal resection of primary cancer at the Department of Surgical Oncology, Nagasaki University Graduate School of Biological Sciences. Among them, 215 patients were diagnosed with Stage III CRC by pathological findings, and 147 of them underwent AC. Although neo-adjuvant chemotherapy (NAC) is usually given to patients with locally advanced colorectal cancer, in the present study, 11 NAC patients were excluded to avoid its confounding effects on AC. Finally, 136 patients were selected for this study. These patients were divided into two groups: the high CAR group (H-group), whose CAR scores were ≥ 0.1, n = 30; and the low CAR group (L-group), whose CAR scores were < 0.1, n = 106).

Before surgery, the appropriateness of resection was determined by abdominal CT and colonoscopy. The following data were collected retrospectively: age, sex, performance status, operation time, amount of blood loss, and postoperative data, including pathology, lymphatic and vessel invasion, histological type, depth of tumor invasion, hospital stay, and 30-day morbidity. AC side effects were graded according to the Common Terminology Criteria for Adverse Events v4.0 (CTCAE) classification, categorizing adverse events from grade 1 to 5 based on the invasiveness of the treatment required. In the present study, adverse events were defined as conditions that required treatment (CTCAE classification Grades 2–5), and CTCAE grade over 3 was defined as a severe side effect.

Colectomy, anterior resection, and abdominoperineal resection plus lymph node resection were performed according to the guidelines of the Japanese Society for Cancer of the Colon and Rectum. A hand-sewn anastomosis or an end-to-end anastomosis using a double stapling technique was performed according to tumor location. Mortality and morbidity data were collected from the database of our department and that of collaborating hospitals.

AC was started within 4 to 8 weeks after surgery. For AC, 5-fluorouracil, TS-1, and capecitabine were used as single agents. Oxaliplatin, 5-fluorouracil, and folinic acid (FOLFOX), S-1 and oxaliplatin (SOX), and capecitabine plus oxaliplatin (XELOX) were selected as combination therapy. Discontinuation was defined as ‘stopped adjuvant chemotherapy during the planning period for any reason’.

### Scoring systems evaluated

The CAR was calculated as serum CRP level (mg/dl)/serum albumin level (g/dl). The GPS is established using the following formula: patients with both hypoalbuminemia (<3.5 g/dl) and an elevated level of CRP (>1.0 mg/dl) were allocated a score of 2. Patients with only one of the two abnormalities were allocated a score of 1. Patients with neither of the above two abnormalities were allocated a score of 0. The PLR is defined as the absolute platelet count divided by the absolute lymphocyte count. The NLR was calculated as the absolute neutrophil count divided by the absolute lymphocyte count. The optimal cut-off levels for CAR, PLR, and NLR were determined by receiver operating characteristic (ROC) curve analyses. All of these laboratory parameters were collected preoperatively, as previously reported [18].

### Statistical Analysis

Data of the different groups were compared using Student’s *t*-test. Continuous data are expressed as means ± standard deviation (SD). On univariate analysis, comparisons of categorical variables were performed using Fisher’s exact test. P<0.05 was considered significant. Overall survival and disease-free survival were calculated according to Kaplan-Meier methods. The differences between groups were tested for significance using the log-rank test. Statistical analysis was performed using SPSS ver.22 (Chicago, IL, USA). Multivariate logistic analysis was used to determine significant factors predicting severe side effects of AC. The factors were selected by the backward elimination method. ROC curves were plotted to identify cut-off values of the CAR (0.1), PLR (147), and NLR (2.4) for side effects. The area under the ROC curve (AUC) was used to assess the power of a model to identify patients who experienced postoperative complications. The AUC values ranged from 0.5 to 1.0, and the greater the AUC, the better the model.

## Results

### Clinicopathological and surgical features and parameters

Table 1 shows the clinicopathological characteristics of each group (H-group and L-group). Age, sex, body mass index, performance status, comorbidities, tumor type, tumor size, tumor depth, lymph node metastasis, and vessel invasion were not significantly different between the two groups. There were significant differences in tumor location (p = 0.04), histological type (p = 0.05), and lymphatic invasion (p = 0.02).

**Table 1:** Clinicopathological characteristics of patients stratified by the CAR.

	CAR<0.1	CAR≥0.1	P-value
**N**	**106**	**30**	
**Age (y)**	**63.8**	**62.4**	**0.50**
**Sex (male/female)**	**59/47**	**20/10**	**0.28**
**Body mass index (kg/m**^**2**^**)**	**22.6**	**23.1**	**0.12**
**Comorbidity (no/yes)**	**74/32**	**18/12**	**0.31**
**Performance status (0/1/2/3)**	**85/14/4/3**	**21/4/4/1**	**0.27**
**Location (C/A/T/D/S/R)**	**4/10/7/5/43/37**	**5/4/3/1/14/3**	**0.04**
**Tumor type (0/1/2/3/4/5)**	**5/8/77/12/4**	**0/3/22/5/0**	**0.51**
**Tumor size (mm)**	**69 (9–103)**	**47 (9–87)**	**0.36**
**T Stage (T1/T2/T3/T4)**	**4/15/73/14**	**1/1/21/7**	**0.27**
**Lymph node metastasis (N1/2/3)**	**66/27/13**	**17/10/3**	**0.68**
**Histological type (well/mod/poor)**	**17/81/8**	**10/16/4**	**0.05**
**Lymphatic invasion (0/1/2/3)**	**4/38/54/10**	**2/8/5/5**	**0.02**
**Vessel invasion (0/1/2/3)**	**13/39/45/9**	**4/10/14/2**	**0.99**

CAR, CRP to albumin ratio.

Table 2 shows the surgical and scoring characteristics of the patients stratified by the CAR. Operation time, blood loss, rate of laparoscopic surgery, postoperative complications, hospital stay, type of AC (single/combined), and PLR score were not significantly different between the two groups.

**Table 2:** Surgical and scoring characteristics of patients stratified by the CAR.

	CAR<0.1	CAR≥0.1	P-value
**Operation time (min)**	**261 (60–713)**	**225 (118–514)**	**0.19**
**Blood loss (g)**	**163 (5–1149)**	**214 (30–1400)**	**0.26**
**Laparoscopic surgery (no/yes)**	**47/59**	**17/13**	**0.23**
**Postoperative complications (no/yes)**	**88/18**	**23/7**	**0.42**
**Hospital stay (days)**	**25.7**	**25.5**	**0.93**
**Chemotherapy (single/combined)**	**69/37**	**19/11**	**0.67**
**Severe side effects (no/yes)**	**87/19**	**14/16**	**<0.01**
**Discontinued (no/yes)**	**91/15**	**20/10**	**0.02**
**NLR**	**2.21 (0.31–7.57)**	**3.03 (0.03–6.83)**	**0.02**
**GPS (0/1/2)**	**99/7/0**	**13/12/5**	**<0.01**
**PLR**	**0.015 (0.004–0.072)**	**0.019 (0.002–0.051)**	**0.07**

CAR, CRP to albumin ratio; NLR, neutrophil to lymphocyte ratio; GPS, Glasgow prognostic score; PLR, platelet to lymphocyte ratio.

In the H-group, more patients experienced severe side effects of AC (p<0.01) and discontinued it (p = 0.02). In regard to the correlations with other scoring systems, there were significant differences for GPS (p<0.01) and NLR (p = 0.02).

### Kaplan-Meier curves of the effect of the CAR on disease-free survival and overall survival

**Fig 1:**
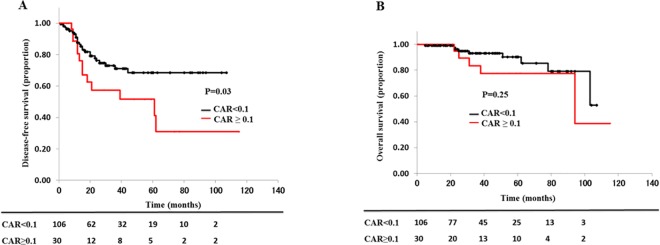
Kaplan-Meier curves of the effect of the CAR on DFS and OS. Disease-free survival is better in the L-group than in the H-group (p = 0.03). However, there is no significant difference in overall survival between the two groups (p = 0.25) (Fig 1B). CAR, CRP to albumin ratio; OS, overall survival; DFS, disease-free survival.

Disease-free survival was better in the L-group than in the H-group (p = 0.03) (Fig 1A). However, there was no significant difference in overall survival between the two groups (p = 0.25) (Fig 1B).

### Severe side effects of chemotherapy and associated parameters

The patients were subdivided into two groups by the severity of side effects of AC (severe side effect group (≥ grade 3), n = 35; mild side effect group (< grade 3), n = 101). Of the 35 severe side effects, 15 were neutropenia, 6 were anorexia, 5 were diarrhea, 2 were hyperbilirubinemia, and anaphylaxis, perforation, acute leukoencephalopathy, liver dysfunction, hand-foot syndrome, general fatigue, and pneumonia accounted for one each (S1 Table).

There were no significant differences in age, sex, performance status, histological type, and postoperative complications. The group who underwent laparoscopic surgery had fewer adverse events (p = 0.01). Combination chemotherapy was correlated with severe side effects (p = 0.002). In the severe side effects group, 21 (60%) patients received combination therapy with SOX 3, FOLFOX 12, or XELOX 6, while the mild side effect group (27, 26.7%) received SOX 11, FOLFOX 10, or XELOX 11 (S2 Table). A high level of the GPS and CAR tended to be associated with severe side effects (p = 0.04, p<0.01; respectively) (Table 3). Multivariate analysis using the clinicopathological factors that were selected using the backward elimination method identified CAR**≥**0.1 (hazard ratio [HR]: 7.06, 95% confidence interval [CI]: 2.51–19.88, p<0.01) and combination chemotherapy (hazard ratio [HR]: 4.94, 95% confidence interval [CI]: 2.01–12.14, p<0.01) as significant determinants of severe side effects of AC (Table 4).

**Table 3:** Univariate analysis of severe side effects during adjuvant chemotherapy.

	Side effects ≥ Grade 3	Side effects < Grade 3	P-value
**n**	**35**	**101**	
**Age (<65/≥65) (y)**	**14/21**	**51/50**	**0.37**
**Sex (male/female)**	**18/17**	**61/40**	**0.35**
**Performance status (0/1/2/3)**	**25/4/4/2**	**81/14/4/2**	**0.27**
**Location (C/R)**	**29/6**	**69/32**	**0.09**
**Tumor size (<30/≥30) (mm)**	**11/24**	**25/76**	**0.44**
**Laparoscopic surgery (no/yes)**	**22/13**	**40/61**	**0.01**
**Combined resection (no/yes)**	**34/1**	**94/7**	**0.37**
**T Stage (T1/T2/T3/T4)**	**0/3/26/6**	**4/13/68/16**	**0.49**
**Histological type (well/mod/poor)**	**14/16/5**	**35/59/7**	**0.39**
**Lymph node metastasis (N1/2/3)**	**20/10/5**	**36/45/20**	**0.05**
**Lymphatic invasion (0/1/2/3)**	**1/15/16/3**	**4/32/53/12**	**0.50**
**Vessel invasion (0/1/2/3)**	**4/12/15/4**	**13/3/44/7**	**<0.001**
**Postoperative complications (no/yes)**	**29/6**	**85/16**	**0.85**
**Chemotherapy (single/combined)**	**14/21**	**74/27**	**0.002**
**Discontinue (no/yes)**	**20/15**	**91/10**	**<0.001**
**CAR(<0.1/≥0.1)**	**20/15**	**87/14**	**<0.001**
**GPS(0/1,2)**	**25/10**	**87/14**	**0.04**
**NLA(<2.4/≥2.4)**	**18/17**	**59/42**	**0.47**
**PLR(<147/≥147)**	**19/16**	**61/40**	**0.52**

CAR, CRP to albumin ratio; NLR, neutrophil to lymphocyte ratio; GPS, Glasgow prognostic score; PLR, platelet to lymphocyte ratio.

**Table 4:** Multivariate logistic regression analysis of grade 3 or 4 side effects during adjuvant chemotherapy.

	HR	95% CI	P-value
**Tumor size (<30 vs. ≥30 mm)**	**0.51**	**0.19–1.36**	**0.19**
**Combination chemotherapy (no vs. yes)**	**4.94**	**2.01–12.14**	**<0.001**
**CAR (<0.1 vs. ≥0.1)**	**7.06**	**2.51–19.88**	**<0.001**

HR, hazard ratio; CI, confidence interval; CAR, CRP to albumin ratio

### Comparison among CAR and other scoring systems

**Fig 2:**
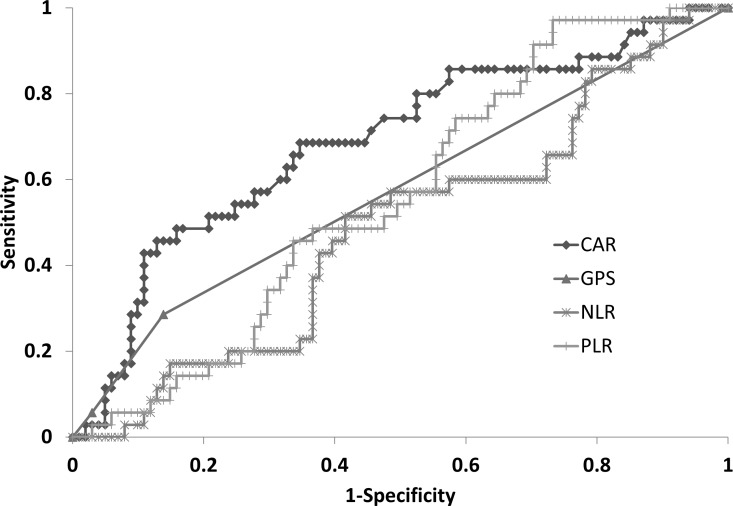
Receiver operating curve analysis for severe side effects. Areas under the curves of the CAR, GPS, PLR, and NLR are 0.79, 0.57, 0.56, and 0.49, respectively. CAR has the highest AUC level among the several inflammation-based scores. CAR, CRP to albumin ratio; NLR, neutrophil to lymphocyte ratio; GPS, Glasgow prognostic score; PLR, platelet to lymphocyte ratio.

On ROC curve analysis, CAR = 0.1 was calculated as the cut-off value for the CAR score to predict severe side effects. The AUCs of each model for the detection of severe side effects of AC were as follows: CAR 0.79, GPS 0.57, PLR 0.56, and NLR 0.49 (Fig 2). The CAR had the highest AUC level of the inflammation-based scores.

## Discussion

It has been reported that AC has a survival benefit compared with surgery alone in CRC patients [5]. Furthermore, combination therapy including oxaliplatin improves both relapse-free survival and overall survival in node-positive CRC [5]. In the 1990s, the rate of AC was relatively small, ranging from 24% to 48% [19]. Recently, Ko and colleagues examined 810 CRC patients with lymph node metastases [20]. They found that 603 patients (74%) received AC postoperatively. Furthermore, more patients received combination therapy (FOLFOX, 59%) than single agent (5-FU, 41%) therapy. In the present study, 215 patients were diagnosed with Stage III, and 147 patients (68%) underwent AC. Combination therapies were selected in 50 patients (34%), with 97 patients given single agents (66%).

During AC, a percentage of patients sometimes experience side effects of chemotherapy. It has been reported that 13% of patients who received 5-FU monotherapy experienced side effects over grade 3 [21]. In the present study, 86 patients received monotherapy, and 13 patients (15%) had severe side effects, which was almost the same as the previous study.

Several clinical trials showed that combination therapy with oxaliplatin had more side effects than 5-FU monotherapy. Kuebler and colleagues reported that severe digestive symptoms such as diarrhea, nausea, vomiting, and dehydration had occurred with oxaliplatin [6]. In the MOSAIC trials, neutropenia, diarrhea, and vomiting were the most frequent grade 3 or 4 adverse effects in the group given combination therapy [5]. They also reported that 74.7% of patients in the group given combination therapy and 86.5% in the monotherapy group had completed AC. Recently, Nakanishi and colleagues examined 169 patients with node-positive CRC; 116 patients received 5-FU monotherapy, and 53 patients received chemotherapy with oxaliplatin, and there were no significant differences between the two groups in tolerability and the completion rate [22]. In the present study, more of the severe side effects group received combination chemotherapy than the low-grade side effects group (60% vs 26.7%) (S2 Table), and multivariate analysis also identified combination chemotherapy as a significant determinant of severe side effects (hazard ratio [HR]: 4.94, 95% confidence interval [CI]: 2.01–12.14, p<0.01), similar to the previous report described above.

A recent study showed that the CAR was a good predictor of the outcome of patients with several types of cancer [11, 12]. Indeed, in the present study, univariate analysis showed that the high CAR group was more likely to have shorter disease-free survival. On Cox regression analysis, CAR status was an independent prognostic factor for disease-free survival (S3 Table).

The CAR consists of CRP and albumin, and both of them are closely correlated with inflammation status. In the state of chronic inflammation, several cytokines could cause weight loss and malnutrition, which could result in severe side effects during chemotherapy [16, 17]. Indeed, the present study demonstrated that high CAR levels were correlated with grade 3 or 4 adverse events during AC on multivariate analysis. Contrary to the previous report, in the present study, the CAR score was not correlated with patient prognosis. Furthermore, the cut-off value of the CAR was 0.1, compared with a score that ranged from 0.02 to 0.038 in previous studies [11, 12, 23]. That may be because, in the present study, node-positive patients were selected, and they may have more inflammation than early-stage patients. In the present study, there were significant differences in tumor location (p = 0.04), histological type (p = 0.05), and lymphatic invasion (p = 0.02) between the high CAR and low CAR groups. These results support previous reports in regard to tumor location and lymphatic invasion [23]. However, the results for histological type were not the same as in a previous colorectal cancer report [12]. This may be a limitation for the setting of a CAR cut-off value.

To the best of our knowledge, no previous report has examined the correlations between inflammation-based scores and the side effects of chemotherapy. In the present study, the relationships of other inflammation-based scores, including GPS, NLR, and PLR, were also examined.

Similar to the CAR, the GPS score is calculated from the CRP and albumin levels, which have been demonstrated to be of prognostic value in several solid cancers [24]. In colorectal cancer, Choi et al. reported that patients with a high GPS score had poorer cancer-specific survival than those with a low GPS score [25].

The PLR and NLR levels are also well-known inflammation-based prognostic systems [26–28]. A high PLR level is reported to be associated with reduced OS and decreased time to recurrence in colorectal cancer patients [29]. It has also been reported that NLR is closely correlated with postoperative complications and prognosis [30, 31].

In the present study, only two scores, the CAR and GPS, were significantly higher in the severe side effect group on univariate analysis (p<0.001, p = 0.04; respectively). Furthermore, multivariate analysis also identified CAR>0.1 (hazard ratio [HR]: 7.06, 95% confidence interval [CI]: 2.51–19.88, p<0.01) as a significant determinant of adverse events (Table 3). These results could facilitate the choice of chemotherapy agents and additional treatment for adverse events before starting AC, which could provide patients with a better quality of life. The AUCs of each model for the detection of severe side effects of AC was highest for the CAR compared to the other inflammatory-based scores (Fig 2). There is a fundamental difference between the CAR/GPS and the PLR/NLR. The CAR and GPS are based on two protein parameters, the serum levels of CRP and albumin. However, the PLR and the NLR consist of two cellular components. Though both the CAR and the GPS use the same components, their results have different implications. The CAR is a simple ratio, regarded as a quantitative variable with a continuous value. However, the GPS is evaluated based on a three-point score and is considered to have a qualitative nature with discontinuous values. Such differences might affect the results of this study.

There were some limitations in this study. First, the number of patients was relatively small. Second, this was a retrospective, single-institution study. The external validation cohort is crucial to assess the potential of the CAR as a predictor for side effects of adjuvant chemotherapy. Third, the proportion of combination therapies was relatively small (35%) compared to a previous study (59%) of node-positive colorectal cancer patients. This is because the selection of ACs was done by two different attending doctors, and they might have chosen 5-FU monotherapy based on a patient’s condition, performance, and wishes. This could have affected the discontinuation rate.

## Conclusion

The present study showed that the CAR is a novel and promising inflammatory-based score for predicting grade 3 or 4 side effects of AC in node-positive CRC patients. Further large-scale studies and analyses using strict criteria are needed.

## Supporting Information

S1 Table Details of side effects greater than grade 3. (PDF)

S2 Table Side effect grades and type of chemotherapy. (PDF)

S3 Table Cox regression analysis of prognostic factors for disease-free survival. (PDF)
