# Supplementary material for: The C-Reactive Protein to Albumin Ratio as a Predictor of Severe Side Effects of Adjuvant Chemotherapy in Stage III Colorectal Cancer Patients
Source: PLoS One. 2016 Dec 8;11(12):e0167967. doi: 10.1371/journal.pone.0167967 (PMC5145220; doi:10.1371/journal.pone.0167967)
Supplement: S1 Table — (PDF) [file pone.0167967.s001.pdf]

Supplementary Table 1: Details of side effects greater than grade 3

**Signs and symptoms**		
	**Neutropenia**	**15**
	**Anorexia**	**6**
	**Diarrhea**	**5**
	**Hyperbilirubinemia**	**2**
	**Anaphylaxis**	**1**
	**Perforation**	**1**
	**Acute leukoencephalopathy**	**1**
	**Liver dysfunction**	**1**
	**Hand-foot syndrome**	**1**

**General fatigue**	**1**
**Pneumonia**	**1**
