# Supplementary material for: The C-Reactive Protein to Albumin Ratio as a Predictor of Severe Side Effects of Adjuvant Chemotherapy in Stage III Colorectal Cancer Patients
Source: PLoS One. 2016 Dec 8;11(12):e0167967. doi: 10.1371/journal.pone.0167967 (PMC5145220; doi:10.1371/journal.pone.0167967)
Supplement: S2 Table — (PDF) [file pone.0167967.s002.pdf]

Supplementary Table 2: Side effect grades and type of chemotherapy

	≥ Grade 3	< Grade 3
**n**	**35**	**101**
**Combination therapy**	**21 (60%)**	**27 (26.7%)**
**Monotherapy**		
**S-1**	**6**	**28**
**UFT**	**6**	**38**
**XELODA**	**2**	**8**
**Combination therapy**		
**SOX**	**3**	**11**
**FOLFOX**	**12**	**10**

**XELOX**	**6**	**6**

UFT, 5-fluorouracil; FOLFOX, oxaliplatin, 5-fluorouracil, and folinic acid; SOX, S-1 and oxaliplatin; XELOX, capecitabine plus oxaliplatin
