# Supplementary material for: The C-Reactive Protein to Albumin Ratio as a Predictor of Severe Side Effects of Adjuvant Chemotherapy in Stage III Colorectal Cancer Patients
Source: PLoS One. 2016 Dec 8;11(12):e0167967. doi: 10.1371/journal.pone.0167967 (PMC5145220; doi:10.1371/journal.pone.0167967)
Supplement: S3 Table — (PDF) [file pone.0167967.s003.pdf]

Supplementary Table 3: Cox regression analysis of prognostic factors for disease-free survival

	HR	95% CI	P-value
**Tumor size (<30 vs. ≥30 mm)**	**0.51**	**0.24-1.08**	**0.07**
**Vessel invasion (0 vs. 1, 2, 3)**	**2.16**	**1.09-4.28**	**0.01**
**NLR (<2.4 vs. ≥2.4)**	**0.16**	**0.29-1.21**	**0.16**
**CAR (<0.1 vs. ≥0.1)**	**4.43**	**1.94-10.15**	**<0.001**

HR, hazard ratio; CI, confidence interval; CAR, CRP to albumin ratio; NLR,

neutrophil to lymphocyte ratio
